# The Effect of Domestication on Inhibitory Control: Wolves and Dogs Compared

**DOI:** 10.1371/journal.pone.0118469

**Published:** 2015-02-25

**Authors:** Sarah Marshall-Pescini, Zsófia Virányi, Friederike Range

**Affiliations:** 1 Comparative Cognition, Messerli Research Institute, University of Veterinary Medicine, Vienna, Medical University of Vienna, University of Vienna, Vienna, Austria; 2 Wolf Science Centre, Ernstbrunn, Austria; University of Padova, ITALY

## Abstract

Inhibitory control i.e. blocking an impulsive or prepotent response in favour of a more appropriate alternative, has been suggested to play an important role in cooperative behaviour. Interestingly, while dogs and wolves show a similar social organization, they differ in their intraspecific cooperation tendencies in that wolves rely more heavily on group coordination in regard to hunting and pup-rearing compared to dogs. Hence, based on the ‘canine cooperation’ hypothesis wolves should show better inhibitory control than dogs. On the other hand, through the domestication process, dogs may have been selected for cooperative tendencies towards humans and/or a less reactive temperament, which may in turn have affected their inhibitory control abilities. Hence, based on the latter hypothesis, we would expect dogs to show a higher performance in tasks requiring inhibitory control. To test the predictive value of these alternative hypotheses, in the current study two tasks; the ‘cylinder task’ and the ‘detour task’, which are designed to assess inhibitory control, were used to evaluate the performance of identically raised pack dogs and wolves. Results from the cylinder task showed a significantly poorer performance in wolves than identically-raised pack dogs (and showed that pack-dogs performed similarly to pet dogs with different training experiences), however contrary results emerged in the detour task, with wolves showing a shorter latency to success and less perseverative behaviour at the fence. Results are discussed in relation to previous studies using these paradigms and in terms of the validity of these two methods in assessing inhibitory control.

## Introduction

Inhibitory control entails blocking an impulsive or prepotent response in favour of a more appropriate alternative when it is advantageous to do so. Inhibitory control mechanisms can help animals enhance their behavioural flexibility, increasing their adaptive success and this may be especially true in unstable or rapidly changing environments, since in these contexts a once advantageous behaviour can quickly become useless or even counterproductive.

Social environments are in many cases unstable since they are made up of social partners and thus require constant negotiation between individual needs whilst maintaining social relationships such that the individual can continue to benefit from inclusion in the group. These challenges may have driven the selection for increased behavioural inhibition as well as other cognitive processes [1–5].

In the context of food competition amongst conspecifics for example, a subordinate may need to inhibit its prepotent response to access resources in order to avoid aggression from higher-ranking individuals and indeed there is some evidence that species with a steeper and more rigid hierarchical organization may have an increased capacity for inhibitory control [6,7]. Further evidence for a link between social factors and behavioural inhibition comes from a study in which primates with a more complex fission-fusion social structure appear to show an increased capacity for inhibitory control over a range of tests [8].

However, fission-fusion is only one of a number of variables characterizing the potential complexity of a species' social structure. A species’ reliance on cooperation in terms of coordinated actions during hunting and/or pair-bonding and communal pup-raising may be another of these variables, since coordinated actions would require inhibitory control to allow for each individual to take into account the behaviour of the other.

In regards to the potential cognitive mechanisms involved in group hunting, in a recent study, authors suggest that an ability to know when to ‘hold back’ vs. when to join, especially when excited may be an important skill that can affect the success of a group hunt (pp.7) [9]. Indeed the timing of actions (or synchronisation) appears to be the main ability required to achieve a ‘collaborative’ hunt as opposed to a hunt in which each individual is striving to obtain the prey at the same time but with no coordinated action.

Pair bonding has also been suggested to have affected a number of cognitive mechanisms with mixed support from experimental studies. In ungulates, carnivores, bats and birds brain size appears to be associated principally with pair-bonding [10], leading authors to suggest that this type of social organization may be associated with complex cognitive skills based on the need to coordinate and synchronize behaviour (pp. 566) [3]. Similarly van Schaik and colleagues [11–13] conclude that pair-bonding and communal pup-raising is indeed associated with a higher performance in socio-cognitive tasks involving (amongst others) the ability to coordinate actions spatially and temporally (e.g. cooperative problem solving). However, based on the current data it is still not clear whether pair-bonding and communal pup-raising positively affect inhibitory control [12].

In terms of their socio-behavioural ecology wolves and free-ranging dogs show similar social organizations in that they can both live in packs and exhibit differential social relationships between members [14–16]. However, the two species appear to differ in their intraspecific cooperation tendencies: in fact although both wolves and dogs rely on close action coordination with pack members when defending their territories [14,17,18], wolves also rely on group coordination when hunting large game [19] whereas reports of group hunting in free-ranging dogs are rare [20] (but see [21]). Furthermore, wolves show pair-bonding and communal raising of pups, with both members of the pair caring for the young and ‘helpers’ (usually offspring from previous years) often staying behind to assist with raising [14, 17]. Female free-ranging dogs, on the other hand, mostly raise their pups alone [22, 23] or in some rare occasions with the help of the male who typically defends but rarely feeds the pups [24]. Hence, based on the limited information we have on the intraspecific socio-behavioural ecology of the two species, one would predict that wolves should show better inhibitory control than dogs.

However, an alternative hypothesis is that through the domestication process, dogs may have been inadvertently selected for a less reactive, "tamer" temperament (‘the emotional reactivity hypothesis’ [25–27]). Indeed ‘the synergistic hypothesis’ of domestication explicitly proposes that compared to wolves, dogs may show superior abilities in certain communicative tasks with humans (e.g. the pointing task) due to the fact that they are more inclined to inhibit their immediate reactions in favour of delayed rewards [28]. Hence, based on these domestication hypotheses, we would expect dogs to have higher inhibitory control than wolves. It is of course possible that both cooperation and domestication have affected inhibitory control, in which case we would expect both species to show an equally developed capacity for inhibition. If this were the case, one could test a third ‘control species’ closely related to dogs and wolves, but with no history of domestication and less dependent on cooperative activities (such as group hunting) to tease these effects apart. Since canids show a high variability regarding socio-ecological variables that may affect cognitive processes such as behavioural inhibition, they are a great model species to investigate such questions. For example, coyotes and jackals rely less on group hunting than grey wolves and Ethiopian wolves tend to be mostly solitary hunters [29].

In the current study, we contribute to such a comparative analysis of inhibitory control in Canids by comparing the performance of identically raised and kept, pack-living wolves and dogs in two inhibitory control tasks. One of these tasks, the cylinder task has already been used to investigate the inhibitory control ability of 36 species, amongst which dogs, wolves and coyotes were tested [30, 31]. Additionally, we presented our subjects with the fence detour task in which pet dogs and dingoes have been tested so far [32–36]. The animals in those studies, however, have been raised under different conditions and had different learning opportunities. Consequently, it is rather difficult to argue for purely evolutionary causes to explain potential differences in results between them, since arguably, training experience may be one of the more influential variables in the development of inhibition. Accordingly, in order to elucidate to what extent inhibitory control is affected by learning and training experiences in wolves and dogs, we furthermore compared the performance of our pack dogs with groups of pet dogs differing in their training experience. Hence the cylinder task was presented also to groups of trained and untrained pet dogs matched for breed and age, and to a group of pet dogs who were given the cylinder test with no prior training trials, to assess whether the initial learning in this phase may affect performance in the test. The performance of wolves on the cylinder task was hence compared to that of dogs living at the Wolf Science Center (WSC) but also to pet dogs with different experiences, whereas performance on the detour task was compared to results of prior studies using this paradigm [32–36].

## Methods

### Ethical statement

No special permission for use of animals (wolves) in such socio- cognitive studies is required in Austria (Tierversuchsgesetz 2012—TVG 2012). The relevant committee that allows running research without special permissions regarding animals is: Tierversuchskommission am Bundesministerium für Wissenschaft und Forschung (Austria).

Ethical approval for the study with pet dogs at the Clever dog lab was obtained from the ‘Ethik und Tierschutzkommission’ of University of Veterinary Medicine (Protocol number 08/04/97/2013) and owner consent was obtained prior to testing each dog. The studies at the WSC were conducted before affiliation with the University of Veterinary Medicine established the need for approval from the ‘Ethik und Tierschutzkommission’ and thus no such ethical approval was obtained.

The Wolf Science Center is located in the game park Ernstbrunn (License No.: AT00012014). The Cites permits for our animals are: 2008: Zoo Herberstein, Austria: AT08-B-0998, AT08-B-0996, AT08-B-0997; 2009: Zoo Basel, Switzerland: AT09-E-0061, Triple D Farm, USA: AT09-E-0018; 2010: Parc Safari, Canada: AT10-E-0018; 2012: Minnesota Wildlife Connection, USA: 12AT330200INEGCJ93, Haliburton Forest, Canada: AT12-E0020.

### Subjects

**WSC pack-dogs and wolves.** Overall 16 wolves (6 F, 10 M) and 14 mixed-breed dogs (7 F, 7 M) housed at the Wolf Science Center were tested with both tasks but 4 individuals (2 wolves and 2 dogs), carried out only one of the two tasks (see Table 1 for details). Furthermore, one dog (Rafiki) had to be excluded from the analyses of the detour task due to a procedural error during testing (the door was not locked properly and the dog succeeded in obtaining the reward in this manner).

**Table 1:** Wolves and pack-dogs tested at the WSC on the detour and cylinder task.

Subject	Dog-Wolf	Sex	Cylinder task	Detour
Kenai	Wolf	M	y	y
Wapi	Wolf	M	y	y
Una	Wolf	F	y	y
Kay	Wolf	F	y	y
Apache	Wolf	M	y	y
Cherokee	Wolf	M	y	y
Geronimo	Wolf	M	y	y
Yukon	Wolf	F	y	y
Nanuk	Wolf	M	y	y
Tatonga	Wolf	F	y	y
Aragorn	Wolf	M	y	y
Kaspar	Wolf	M	y	y
Shima	Wolf	F	y	y
Tala	Wolf	F	y	y
Amarok	Wolf	M	n	y
Chitto	Wolf	M	n	y
Bora	Dog	F	y	y
Layla	Dog	F	y	y
Nia	Dog	F	y	y
Nuru	Dog	M	y	y
Zuri	Dog	F	y	y
Meru	Dog	M	y	y
Asali	Dog	M	y	y
Bashira	Dog	F	y	y
Binti	Dog	F	y	y
Hakima	Dog	M	y	y
Kilio	Dog	M	y	y
Maisha	Dog	M	y	n
Rafiki	Dog	M	y	y but error
Alika	Dog	F	n	y

The detour task was presented to animals when they were 10 months of age, whereas the cylinder task was presented to animals between 9 months and 3 years of age (balancing age across groups: wolves: mean 1.7 years; dogs mean SD 2.1 years).

Dogs and wolves at the WSC (http://www.wolfscience.at) are raised and kept in the same way, establishing long-term close bonds with the trainers who care and work with the animals on a daily basis. In particular, all animals are trained to wear a collar and the standard procedure for many tests requires animals to sit or stand whilst being held by the collar by their trainers. All animals participate in various behavioural tests every week, where they are rewarded with food. All dogs and wolves are worked in separation from their pack members on a daily basis and participation in all training and testing sessions are voluntary. The trainers, as the people having daily contact with the animals in many different contexts (e.g. training, testing, leash walks, standard physical veterinary care etc.) and in most cases having raised them from puppyhood have a very close relationship with the animals, the latter being very comfortable and at ease around them. For more details relating to the upbringing and keeping of the animals please see [37,38].

### Pet dogs

In addition, 24 age and breed matched (i.e. mixed breed) pet dogs were tested on the cylinder task; 12 dogs regularly carried out sport training activities with their owner (7F; 5M,) and 12 (6F; 6M) had either no or just basic training experience (owner-trained to perform the basic heel, sit, down commands). Finally, 13 (8F; 5M) pet dogs were tested with a modified version of the cylinder task, in which no training phase was presented (see procedure below). In accordance with the testing procedure at the Wolf Science Center, all pet dogs were tested in an outdoor enclosure at the Vetmed, Clever Dog Lab.

## Experiment 1- The Detour Task

### Apparatus

We used a 1 m high V-shaped fence, the sides of which were 3 m long and closed at an angle of 80°. Each side consisted of 3 identical sections constructed of a wooden frame covered by a wire mesh. Based on [36], two swing-doors (0.4 x 0.4 m) were mounted into the front, lower section of both sides of the fence and could be opened upwards and fixed in either an open or a closed position.

### Experimental conditions

In the experiment, the subjects had to retrieve a food reward (a dead, one day old chick) by detouring along the fence. Due to the relatively small sample size, we used a within-subject design, with each animal being tested in three 60 second long trials that were started one after the other with short (1–3 min) inter-trial intervals.

The three trials consisted of two different conditions:

Trial 1: doors were closed; animals had to detour to get the food reward (condition 1)

Trial 2: doors were open; animals could directly access the food reward through the open doors (condition 2)

Trial 3: doors were closed (condition 1)

The experiment was videotaped from a distance either by an assistant or by positioning the camera on a tripod.

### Experimental procedure

Before testing started, the experimenter walked around the sides (including the inner sides) of the fence 3 times in both directions whilst being invisible to the subject. The start position was set at 3–4 m from the intersecting angle of the fence. The subject was led to the start position on a leash by a familiar experimenter. A second familiar experimenter showed the food reward to the subject and then, calling the attention of the subject and showing the food reward, went to the intersecting angle of the fence and dropped the reward on the inner side of the fence. After returning to the starting position and showing her empty hands, the subject was released. Once the subject was released, the two experimenters remained in the starting position and did not communicate with the subject in any way. A trial was terminated if the subject retrieved the reward or after 60 seconds. The subject was then called back to the starting position.

### Coding and analysis

From the test videos, we extracted the latency to find the chick starting when the animal was unleashed after the demonstrator had returned to the starting position and showed her empty hands. Furthermore, we coded the total duration that any part of the subject (excluding the tail) remained within 30 cm of the first segment of the fence as a measure of a lack in inhibitory control e.g. not being able to move away from the food on the other side of the fence.

Twenty percent of trials were coded by a second person, and Spearman correlations were calculated trial by trial for both the latency to obtain the reward and the duration of spending time at the fence (all correlation coefficients were above 0.91).

Mann-Whitney U tests were carried out to compare performance between dogs and wolves in the test trials and Wilcoxon matched-pairs signed-ranks tests when comparing performance of each species across trials. Coding was conducted using Solomon Coder beta (© András Péter). Statistical analyses were carried out using SPSS version 19.

## Results- Detour Task

**Fig 1:**
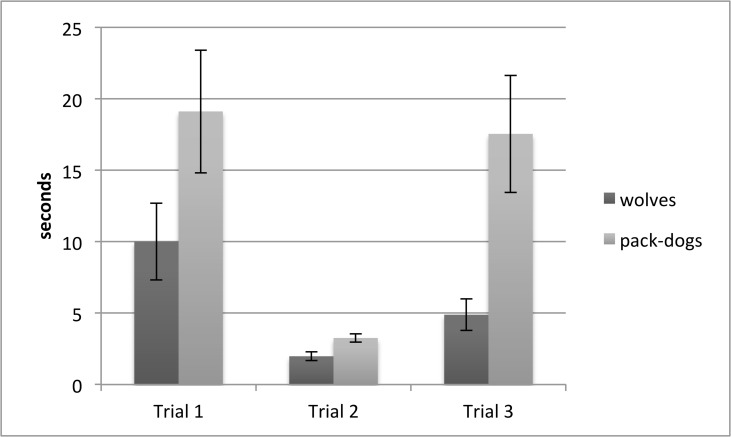
Wolves’ and pack-dogs’ performance in the detour task. Mean duration (in seconds) and SE of time spent close to the fence in front of the food by wolves and dogs in the detour task. *p<0.01.

In the first trial, wolves succeeded in detouring the fence significantly faster than the dogs (mean±SE: wolves: 22.41±4.35, dogs: 34.13 ± 5.7; U = 51.0, p = 0.037), with two dogs failing to detour within the 60 second trial time. The slower detouring as well as the failing of the dogs can be explained by spending more time at the fence in proximity to the food e.g. showing less inhibitory control than the wolves (mean±SE: wolves: 10.01 ± 2.73, dogs: 19.12 ± 4.3; U = 44, p = 0.016) (Fig. 1).

In the second trial, wolves and dogs differed from each other neither in the latency (mean±SE: wolves: 4.53 ± 0.65, dogs: 3.29 ± 0.27; U = 85.0, p = 0.416) nor the time they spent close to the fence (mean±SE: wolves: 1.98 ± 0.34, dogs: 3.26 ± 0.29; U = 115.5, p = 0.364). However, four wolves never used the shortcut through the open doors but went straight to detouring the fence again, whereas all dogs used the shortcut.

In the third trial, wolves and dogs once again differed from each other in the latency with wolves being faster (mean±SE: wolves: 9.69 ± 1.18, dogs: 32.62 ± 7.16; U = 21.5, p = 0.001) and dogs spending more time close to the fence in front of the food than wolves (mean±SE: wolves: 4.89 ± 1.14, dogs: 17.55 ± 4.13; U = 36.0, p = 0.005). In this third trial, both the dogs that had failed in the first trial and an additional, previously successful dog failed to detour within the 60 second trial time.

Finally, while the wolves became significantly faster in detouring the fence from trial 1 to trial 3 (T+ = 1.5, p = 0.001), and also showed better inhibitory control in that they spent less time at the fence in front of the food, (T+ = 12.5, p = 0.007), this was not the case for dogs (latency: T+ = 27.0, p = 0.594; inhibitory control: T+ = 31.0, p = 0.53).

## Experiment 2- The Cylinder Task

### Apparatus

**Fig 2:**
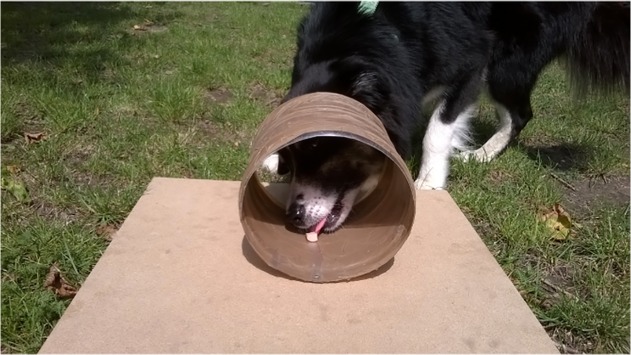
The opaque cylinder used in training trials. The exact same apparatus, but transparent, was used in test trials.

The apparatus consisted of a cylinder (22cm in length, 20 cm in diameter) that was open on both sides and attached to a wooden base for support. In training trials, the cylinder was opaque (Fig. 2), while in test trials it was exactly the same but transparent.

### Experimental setup

In general the experimental setup consisted of one person holding the animal by its collar, whilst the other visibly placed food in the apparatus, and then releasing the animal. Upon retrieval of the food, the animal was called back by the first experimenter. This sequence was repeated in both training and test trials. Training and test trials differed only in the fact that the apparatus was opaque in training trials and transparent in test trials.

### Experimental Procedure

Testing took place in a fenced outdoor area at the WSC. Experimenter 1 was one of the trainers, who positioned the animal 1.5 m from the apparatus, facing the opaque cylinder, holding it by the collar. The second experimenter stood behind the cylinder apparatus, showed the subject a treat, called its’ name, placed the treat into the tube and removed her hand, standing up.

Once the experimenter stood up straight, the handler released the animal. After releasing the animal, both the experimenter and the handler remained in the same position. On each trial, the experimenter recorded whether the dog first made a correct or incorrect choice. A choice was coded as “correct” if the animal’s snout entered the open end of the cylinder without the animal first touching the exterior of the cylinder with any part of his or her head or paw. Conversely, a choice was coded as “incorrect” if the animal touched the exterior of the cylinder with its snout or paw prior to finding the treat. In order to move on to the test trials, the animal was required to make a correct first choice in four of five consecutive training trials. If after 10 training trials the animal still had not succeeded in reaching criterion, the test was discontinued and repeated on the following day.

Since test trials were identical to the training trials except that the opaque cylinder was replaced with the transparent cylinder in the former trials, the animals could see the food through the cylinder, introducing a competition between the correct motor response (established during the training trials) and visual input (which could lead the animal to approach the food directly, bumping into the front of the cylinder). As in training trials, the experimenter recorded whether the dog made a correct or incorrect choice on each trial, using the same criteria.

The side from which the experimenter baited the cylinder was counterbalanced across animals. The treat was always placed in the middle of the tube, making it accessible to the animals via either side. All sessions were video-recorded.

The same exact procedure was carried out with untrained and highly trained pet dogs tested at the Clever Dog Lab, but in this case the owner acted as the handler holding the dog by its collar (hence in both studies, the person the animals felt most comfortable with, handled the animal). The final group of pet dogs tested was given the test trials, as described above, but with no training trials. The final group of dogs was tested in this manner to assess the effect of the initial exposure to the opaque apparatus on performance in test trials.

### Coding and analysis

Twenty percent of trials were coded by a second person, and inter-rater reliability was 100%. A Mann-Whitney U test was carried out to compare performance between dogs and wolves in training and test trials, a Wilcoxon test was used to compare performance in the first compared to the last 5 test trials, a Spearman’s correlation was used to correlate training with test trials and a Kruskall-Wallis to compare performance between pack-living dogs, trained and untrained pet dogs and dogs’ that had not had the familiarization phase of the test. SPSS version 19 was used for statistical analyses.

## Results- Cylinder Task

**Fig 3:**
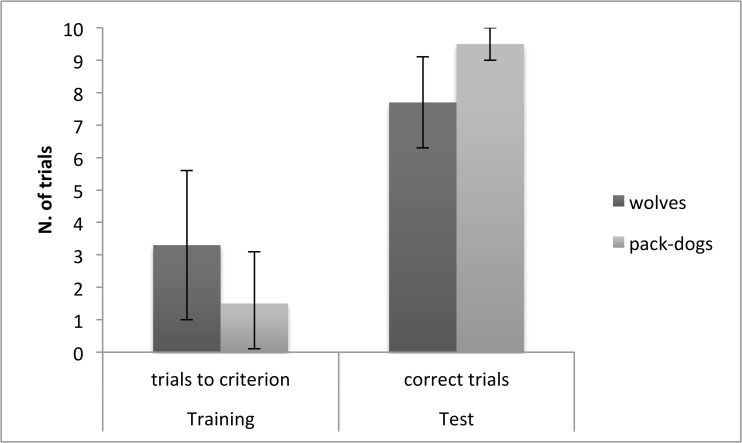
Wolves’ and pack-dogs’ performance in the cylinder task. Mean number (and 95% confidence interval) of trials to criterion in the training phase, and of correct trials in the test phase, for wolves and dogs in the cylinder task. *p<0.01.

No significant differences emerged between identically raised wolves and dogs in the number of training trials required to reach criterion (mean±SE: dogs: 1.7±0.8, wolves: 3.3±1; U = 120, p = 0.13); however, a significant difference was observed in test trials with dogs carrying out significantly more correct responses than wolves (mean±SE: wolves: 7.7±0.2, dogs: 9.5±0.6; U = 41.5, p = 0.01) (Table 2; Fig. 3). On average dogs made correct choices on 95% and wolves on 77% of test trials. No correlation was found between performance in training trials and test trials for neither wolves nor dogs (wolves: R = 0.028, p = 0.92; dogs: R = 0.029, p = 0.92).

**Table 2:** Test scores (correct out of 10) in the cylinder task, sex and age of wolves and dogs tested at the WSC.

Subject	Dog-Wolf	Sex	Age (in years)	Score ‘cylinder task’
Kenai	Wolf	M	0.9	10
Wapi	wolf	M	0.9	8
Una	wolf	F	1.2	10
Kay	wolf	F	1.2	4
Apache	wolf	M	1	7
Cherokee	wolf	M	1.8	9
Geronimo	wolf	M	1.9	2
Yukon	wolf	F	1.9	7
Nanuk	wolf	M	1.9	10
Tatonga	wolf	F	1.9	5
Aragorn	wolf	M	2.9	9
Kaspar	wolf	M	2.9	9
Shima	wolf	F	2.9	9
Tala	wolf	F	1.2	9
Bora	dog	F	1.4	10
Layla	dog	F	1.4	8
Nia	dog	F	1.4	10
Nuru	dog	M	1.5	10
Zuri	dog	F	1.5	9
Meru	dog	M	2.2	10
Asali	dog	M	2.3	10
Bashira	dog	F	2.3	10
Binti	dog	F	2.3	10
Hakima	dog	M	2.3	9
Kilio	dog	M	3	10
Maisha	dog	M	3	8
Rafiki	dog	M	3	10

Wolves’ ‘incorrect’ responses mostly consisted of placing their nose/snout on the transparent tube in correspondence with the food prior to inserting their snout from the side. On a few occasions a wolf would also paw the apparatus prior to obtaining the food from the side. Biting the apparatus was seen very rarely.

No learning effects were observed for neither wolves nor dogs in test trials, in that there was no difference in performance between the first and last 5 test trials (Wilcoxon: wolves: z = 1.8, p>0.05; dogs: z = 1.5, p>0.05).

**Fig 4:**
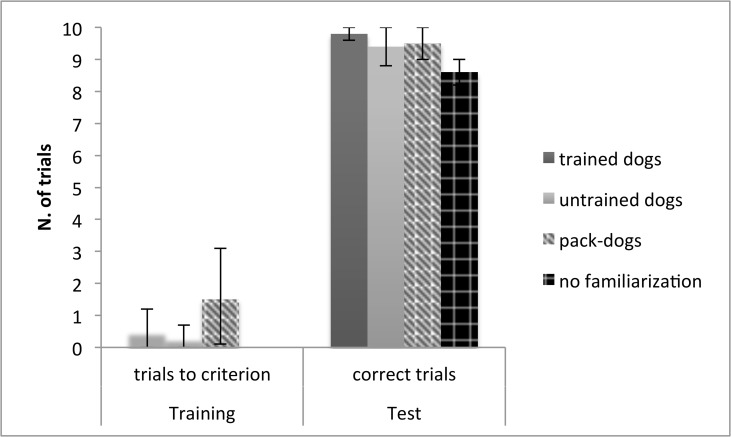
Pet- and pack- dog’s performance in the cylinder task. Mean number (and 95% confidence interval) of trials to criterion in the training phase, and of correct trials in the test phase, for trained and untrained pet dogs and dogs tested with no prior familiarization with the opaque cylinder.

WSC pack-living dogs, trained and untrained pet dogs, and dogs tested with no prior familiarization phase were also compared. No difference emerged between pack-dogs and trained and untrained pet dogs in the number of training trials required to reach criterion (mean±SE: pack-living dogs: 1.7±0.8, trained pet dogs: 0.5±0.3, untrained pet dogs: 0.3±0.2; T+ = 1.3, p = 0.5). Furthermore, no differences emerged between all four groups in the number of correct test trials (mean±SE pack-living dogs: 9.5±0.6, trained pet dogs: 9.8±0.1, untrained pet dogs: 9.4±0.3dogs, no training phase dogs: 8.6±0.6; T+ = 4.8, p = 0.19) (Fig. 4). On average trained dogs made correct choices on 98%, untrained dogs on 94% and dogs tested with no prior training trials on 86% of test trials.

## Results across Experiments

To assess whether the subject’s behavioural inhibition performance correlated across tasks, a Spearman’s test was carried out separately for those wolves and dogs that had carried out both tasks. Results showed no correlational pattern between success in the cylinder task and duration of staying at the fence in neither wolves (N = 14, rho = 0.12, p = 0.68) nor dogs (N = 11, rho = 0.15, p = 0.65), nor a correlation between success in the cylinder task and latency to obtain the reward in the detour task (dogs: rho = 13, p = 0.69; wolves: rho = 0.42, p = 0.14).

## Discussion

Two contrasting predictions were put forward as to the wolves’ and dogs’ performance in an inhibitory control task; one based on the differences in the intraspecific socio-behavioural ecology of the two species, the other based on the potential changes caused by the process of domestication. However, considering wolves outperformed dogs in the first inhibitory control task (the fence detour) but identically-raised pack dogs outperformed wolves in the other inhibitory control task (the cylinder task), it appears that the current results cannot provide clear support for neither one nor the other hypothesis. Results are henceforth discussed for each task separately with a final section addressing the potential reasons for the contradictory findings observed.

### The detour task

Compared to other studies using the same fence detour task, although wolves were faster than pack-dogs in detouring the fence, both performed within the reported mean latency for successful pet dogs (i.e. between 20 and 40 seconds [32–34]) and both were considerably slower than dingoes (reported mean for first trial is 10.25 seconds [35]). Similarly to previous studies, pack-dogs did not show an improvement in their latency to success over consecutive trials [32,33], while wolves, similarly to dingoes, decreased in their latency to success (over trials) [35]. Differently from previous studies, however, we were particularly interested in the detour task as a measure of behavioural inhibition, which, in this task, would be inhibiting the prepotent response of seeking to access the food just in front of the animal. For this reason our comparison included the time spent by animals close to the food source whilst engaged in any activity, from just standing and looking at it, to pawing and scratching the fence. Importantly this measure avoids the potential effect that differences in the body length and speed between wolves and dogs may have on their latency to obtaining the food. But also based on this measure, wolves consistently outperformed dogs, in that they were able to move away from the position immediately in front of the food significantly sooner than dogs suggesting better inhibitory control.

Hence based on these results it would seem that wolves have retained a better capacity to inhibit an ineffectual behaviour in favour of trying out an alternative action. These results are line with observations from another study, which compared dogs and wolves in their ability to socially learn how to access a food box by operating a lever [37]. In this study only a small subsample of dogs successfully opened the apparatus, whereas all wolves did. Interestingly for the current issue, although both wolves and dogs initially approached the functional area of the apparatus where the lever was, dogs were more likely to first manipulate the side or back of the box which allowed direct visual and olfactory access to the food inside. Wolves did not show this preference suggesting that dogs may have found it harder to inhibit the more direct exploration of the food resource and concentrate on exhibiting the more functional behaviours observed (i.e. manipulating the lever).

Overall, based on these results, and contrary to the ‘domestication hypotheses’, we could conclude that the shift in social structure from wolves’ pair-bonded family unit, reliant on all family members for hunting and pup-provisioning, to a multi-male multi-female structure, reliant on more static food sources (e.g. human refuse) and a less intense level of involvement in pup-rearing, may have relaxed dogs’ inhibitory control abilities.

### The cylinder task

Results from the cylinder task show that pack-dogs made significantly fewer mistakes than wolves, showing a more consistent ability to inhibit the prepotent response of going directly for the food, in favour of reaching around to the open mouth of the cylinder. In contrast wolves tended to first put their nose or their paw on the transparent cylinder in correspondence with the visible food, before redirecting their snout to the lateral opening.

One possible explanation for this difference could be that dogs acquire the ‘correct’ response more firmly during training trials, and hence do not ‘fall into’ making mistakes once the transparent tube is presented. However, the performance of a group of pet dogs presented only with test trials and no familiarization trials, shows that the dogs’ high success rate in the test is independent of prior experience with the opaque apparatus. Indeed, even level of training, which has been discussed to influence inhibitory control, did not affect dogs’ performance in this task since no differences emerged between trained and untrained pet dogs (both groups performing like pack-dogs).

The high scores obtained by dogs in our study, contrast to some extent with the only other study using this paradigm in dogs. Indeed the average correct response for dogs in the [30] previous study was 70%, whereas in the current study, dogs of different groups (i.e. pack-dogs and both trained and untrained pet dogs) that had undergone the same exact procedure showed an average correct response of 90%. Most of our dogs (including pack-dogs, and trained and untrained pet dogs) performed at ceiling with 89% (33 out of 37) of individuals scoring either 9 or 10 out of 10. Indeed even dogs that had no prior familiarization training performed at ceiling with 70% (9 out of 13) of individuals obtaining top scores. However, this difference between studies may be due to subject sample differences (breed, neutering status, reward quality) or the fact that dogs in the Bray et al. study [30], carried out the cylinder task after having been tested on another inhibitory control task (MacLean personal communication) since it has been shown for both humans and dogs, inhibitory control may be affected by resource depletion [39]. Furthermore, other methodological differences between studies, such as the visibility of the task and/or the neutering status of the subjects may have further affected results (MacLean personal communication).

The consistently high scores obtained by the dogs nevertheless contrast strikingly with the much less consistent performance of wolves. In their case 57% (8 of a total 14) of the study population scored on a par with dogs’ (9 out of 10 or above), whereas scores for the remaining individuals ranged from 2 to 7 correct trials out of 10. Taken together results suggest that the variability in the wolf population was far greater than that seen in dogs, hinting to the possibility that there may be multiple factor’s affecting wolves’ inhibitory control, influencing their less consistent performance in this task.

Hence based on these results it would seem that dogs consistently outperform wolves on this inhibitory control task, and that their high performance rate is independent of both contextual and more general life experiences. Results showing better inhibitory control in dogs than wolves are in line with previous studies showing that compared to young wolves, young dogs display greater control of agonistic behaviours and inhibition of actions in a food related task [28]. Results would hence provide support to the various versions of the ‘domestication hypotheses’ suggesting that an important change brought about by the domestication process is an increase in inhibitory control [28, 25–27, 40].

However, results from the two tasks presented here, are in direct contradiction with one another, hence one of the main questions is whether the tasks currently used do indeed both measure inhibitory control, and what factors maybe responsible for the contrasting results.

### Do both tasks measure behavioural inhibition?

As shown by the lack of correlation in performance between the two tasks (at the species level) it is not altogether clear whether the cylinder and the detour task measure the same underlying process. Interestingly both tasks have been used as measures of inhibitory control before with a number of different species and both are considered ‘detour’ tasks, in as much as they require animals to orient their movement away from the most direct approach to the food source, however the two tasks have never been used in conjunction.

A number of studies with other species have shown that inhibitory control may be context specific. For example, in a delayed choice task, [41] carried out a series of studies with bonobos, chimpanzees and humans and found that whereas humans were less willing to wait for food rewards than chimpanzees, they were more willing to show patience when the reward was monetary. These results indicate that inhibitory control allows adaptive responses to be present in a variety of decision-making contexts, but that results may vary across these. Indeed, Bray et al. [30] found a similar phenomenon with dogs. The same dogs were tested in three different experimental paradigms involving in one case a social context (where a ‘selfish’ donor with preferred food had to be avoided to obtain a less preferred food from a generous individual), and in the other two tasks a non-social context (where the first task was a A-not-B test where animals had to inhibit searching for food in a previously rewarded location and the second task a cylinder test also presented in the current study). Interestingly, not only was there no correlation in performance between the social and non-social tasks, but there was also none between the non-social ones (the A-not-B and cylinder test). Authors suggest that alongside behavioural inhibition other cognitive mechanisms (e.g. quantity discrimination, physical cognition etc.) may have been involved and that these may affect the performance differently across individuals and tasks.

A similar argument may explain the contradictory results in the current study. The detour task may require, as well as inhibitory control, a level of physical understanding of the spatial relationships between the fence, the object and the subject, and this may vary across species. Indeed early work comparing wolf and dog pups at 6 and 10 weeks showed that wolf pups had a higher performance on a number of tasks relating to physical cognition, amongst which were both a detour-like task (‘the barrier task’) and a maze navigation test [42]. This early work led Frank and colleagues, to suggest that the effect of domestication may have had a detrimental effect on dogs’ physical cognition [43–45]. However, later work on means-end understanding showed no differences between wolves and dogs in this task [46] hence whether domestication has negatively affected dogs’ abilities in the physical cognition domain and spatial abilities in particular remains to be confirmed by future studies.

Similarly, performance on the cylinder task may be affected by the level of attention given to the actions performed by the experimenter, i.e. noticing that the food is placed into the cylinder from the side entrance. Because the filming angle was such that only the behaviour on the apparatus could be observed, unfortunately in the current study we were unable to measure the looking behaviour and the amount of struggling behaviour displayed by the animals whilst being held by the collar during demonstrations. However, as has been shown by a number of studies [28, 47–49] dogs show a greater propensity to gaze at their human partners, and indeed at least in dog and wolf pups, the latter’s decreased attention to the experimenter has been shown to negatively affect performance in pointing tasks [28].

Overall results point to the difficulty in obtaining a ‘clean’ measure of inhibitory control, with no other factors affecting results and suggest that a multi-task approach to evaluating species differences may be necessary before firm conclusions can be drawn on the potential factors affecting self control [31].

## Supporting Information

S1 File Dataset for both the Detour task and Cylinder task. (XLSX)

## References

[1] Byrne RW, Whiten A (1988) Machiavellian Intelligence: Social Expertise and the Evolution of Intellect in Monkeys, Apes and Humans Oxford: Oxford University Press

[2] Dunbar RIM (1998) The social brain hypothesis. Evol Anthropol 6: 178–190.

[3] Dunbar RIM (2009) The social brain hypothesis and its implications for social evolution. Ann Hum Biol 36(5): 562–572. 10.1080/03014460902960289 19575315

[4] Dunbar RIM, Shultz S (2007) Evolution in the social brain. Science 317: 1344–1347. 17823343 10.1126/science.1145463

[5] Byrne RW, Bates LA (2007) Sociality, evolution and cognition. Curr Biol 17: 714–723. 10.1016/j.cub.2007.05.069 17714665

[6] Thierry B (2007) Unity in diversity: lessons from macaque societies. Evol Anthropol 16: 224–238.

[7] Amici F, Call J, Aureli F (2009) Variation in withholding of information in three monkey species. Proc R Soc B 276: 3311–3318. 10.1098/rspb.2009.0759 19535370 PMC2817172

[8] Amici F, Aureli F, Call J (2008) Fission-Fusion Dynamics, Behavioral Flexibility, and Inhibitory Control in Primates. Curr Biol 18: 1415–1419. 10.1016/j.cub.2008.08.020 18804375

[9] Bailey I, Myatt JP, Wilson AM (2013) Group hunting within the Carnivora: physiological, cognitive and environmental influences on strategy and cooperation. Behav Ecol Sociobiol 67: 1–17.

[10] Shultz S, Dunbar RIM (2007) The evolution of the social brain: anthropoid primates contrast with other vertebrates. Proc Roy Soc Lond B 274: 2429–2436. 10.1098/rspb.2007.0693 PMC2274976 17652066

[11] Burkart JM, Hrdy SB, van Schaik CP (2009) Cooperative breeding and human cognitive evolution. Evol Anthropol 18 (5): 175–186.

[12] Burkart JM, van Schaik (2010) Cognitive consequences of cooperative breeding in primates. Anim Cogn 13(1): 1–19. 10.1007/s10071-009-0263-7 19629551

[13] van Schaik CP, Burkart JM (2009) Mind the gap: cooperative breeding and the evolution of our unique features. In: Kappeler PM, Silk J, editors. Mind the gap: tracing the origins of human universals. pp. 477–496.

[14] Mech LD, Boitani L (2003) Wolf Social Ecology In: Wolves: Behavior, Ecology, and Conservation Mech LD, Boitani L, editors. pp. 1–35. Chicago, London: The University of Chicago Press.

[15] Cafazzo S, Valsecchi P, Bonanni R, Natoli E (2010) Dominance in relation to age, sex and competitive contexts in a group of free-ranging domestic dogs. Behav Ecol 21: 443–455.

[16] Bonanni R, Cafazzo S, Valsecchi P, Natoli E (2010) Effect of group size, dominance rank and social bonding on leadership behaviour in free-ranging dogs. Anim Behav 79: 981–991.

[17] Mech D (1970) The wolf: the ecology and behaviour of an endangered species Garden City, NY: Natural History Press 10.3109/inf.1970.2.issue-3.14

[18] Bonanni R, Valsecchi P, Natoli E (2010a) Pattern of individual participation and cheating in conflicts between groups of free-ranging dogs. Anim Behav 79: 957–968.

[19] Schmidt PA, Mech LD (1997) Pack size and food acquisition. Am Nat 150:513–517. 10.1086/286079 18811290

[20] Butler JRA, du Toit JT, Bingham J (2004) Free-ranging domestic dogs (*Canis familiaris*) as predators and prey in rural Zimbabwe: threats of competition and disease to large wild carnivores. Biol Conserv 115:369–378.

[21] Manor R, Saltz D (2004) The impact of free-roaming dogs on gazelle kid/female ratio in a fragmented area. Biol Conserv 119: 231–236.

[22] Boitani L, Ciucci P (1995) Comparative Social Ecology of Feral Dogs and Wolves. Ethology Ecology & Evolution 7:49–72. 10.1371/journal.pone.0115814 25615596 PMC4304801

[23] Daniels TJ, Bekoff M (1989) Population and social biology of free-ranging dogs, *Canis familiaris* . Journal Mammal 70: 754–762.

[24] Pal SK (2005) Parental care in free-ranging dogs, *Canis familiaris* . Appl Anim Behav Sci 90: 31–47.

[25] Hare B, Tomasello M (2005) Human-like social skills in dogs? Trends Cogn Sci 9: 439–444. 16061417 10.1016/j.tics.2005.07.003

[26] Hare B, Plyusnina I, Ignacio N, Schepina O, Stepika A, Wrangham R, et al (2005) Social cognitive evolution in captive foxes is a correlated by-product of experimental domestication. Curr Biol 15: 226–230. 15694305 10.1016/j.cub.2005.01.040

[27] Hare B, Wobber V, Wrangham R (2012) The self-domestication hypothesis: evolution of bonobo psychology is due to selection against aggression. Anim Behav 83(3): 573–585.

[28] Gácsi M, Gyori B, Virányi Z, Kubinyi E, Range F, Belényi B, et al (2009) Explaining Dog Wolf Differences in Utilizing Human Pointing Gestures: Selection for Synergistic Shifts in the Development of Some Social Skills. PLoS ONE 4(8): e6584 10.1371/journal.pone.0006584 19714197 PMC2719091

[29] Sillero-Zubiri C, Hoffman M, Macdonald DW (2004). Canids: foxes, wolves, jackals and dogs Status Survey and Conservation Action Plan, vol. 62 IUCN, Gland, Switzerland, and Cambridge, UK.

[30] Bray EE, MacLean EL, Hare BA (2013) Context specificity of inhibitory control in dogs. Anim Cogn 17(1): 15–31. 10.1007/s10071-013-0633-z 23584618 PMC4154138

[31] MacLean EL, Hare B, Nunn CL, Addessi E, Amici F, Anderson RC, et al 2014 The Evolution of Self-control P Natl Acad Sci USA 111(20): e2140–e214. 10.1073/pnas.1323533111 24753565 PMC4034204

[32] Pongrácz P, Miklósi Á, Kubinyi E, Gurobi K, Topál J, Csányi V (2001) Social learning in dogs: the effect of a human demonstrator on the performance of dogs in a detour task. Anim Behav 62: 1109–1117.

[33] Pongrácz P, Miklósi Á, Vida V, Csányi V (2005) The pet dogs’ ability for learning from a human demonstrator in a detour task is independent from the breed and age. Appl Anim Behav Sci 90: 309–323.

[34] Pongrácz P, Vida V, Bánhegyi P, Miklósi Á (2008) How does dominance rank status affect individual and social learning performance in the dog (*Canis familiaris*)? Anim Cogn 7: 90–97. 10.1007/s10071-007-0090-7 17492317

[35] Smith BP, Litchfield CA (2010) How well do dingoes, *Canis dingo*, perform on the detour task? Anim Behav 80: 155–162.

[36] Pongrácz P, Miklósi Á, Kubinyi E, Topál J, Csányi V (2003) Interaction between individual experience and social learning in dogs. Anim Behav 65: 595–603.

[37] Range F, Virányi Z (2013) Social learning from humans or conspecifics: differences and similarities between wolves and dogs. Front Psychol 10.3389/fpsyg.2013.00868 PMC3849518 24363648

[38] Range F, Virányi Z (2013) Wolves are better imitators of conspecifics than dogs. PLoS ONE 10.1371/journal.pone.0086559. PMC3906065 24489744

[39] Miller HC, Pattison KF, DeWall CN, Rayburn-Reeves R, Zentall TR (2010) Self-control without a “self”?: common self-control processes in humans and dogs. Psychol Sci 21(4):534–538. 10.1177/0956797610364968 20424096

[40] Virányi Z, Range F (2014) On the way to a better understanding of dog domestication: aggression and cooperativeness in dogs and wolves In: The Social Dog: behaviour and cognition Kaminski J, Marshall-Pescini S editors. San Diego, CA: Elsevier.

[41] Rosati AG, Hare B (2013) Chimpanzees and Bonobos Exhibit Emotional Responses to Decision Outcomes. PLoS ONE 8(5): e63058 10.1371/journal.pone.0063058 23734175 PMC3667125

[42] Frank H, Frank MG (1987) The University of Michigan Canine Information Processing project (1979–1981) In Man and wolf Frank H, editor. pp. 275–292. Dordrecht, The Netherlands: W. J. Publishers.

[43] Frank H, Frank MG (1982) Comparison of problem-solving performance in six-week-old wolves and dogs. Anim Behav 30:95–98.

[44] Frank H, Frank MG (1985) Comparative manipulation test performance in ten-week-old wolves (*Canis lupus*) and Alaskan malamutes (*Canis familiaris*): A Piagetian interpretation. J Comp Psych 99:266–274.

[45] Frank H, Frank MG, Hasselbach LM, Littleton DM (1989) Motivation and insight in wolf (*Canis lupus*) and Alaskan malamute (*Canis familiaris*): Visual discrimination learning. B Psychonomic Soc 27(5): 455–458.

[46] Range F, Möslinger H, Virányi Z (2012) Domestication has not affected the understanding of means-end connections in dogs. Anim Cog 15(4): 597–607. 10.1007/s10071-012-0488-8 22460629 PMC4306441

[47] Gácsi M, Gyori B, Miklósi Á, Virányi Z, Kubinyi E (2005) Species-Specific Differences and Similarities in the Behavior of Hand-Raised Dog and Wolf Pups in Social Situations with Humans. Dev Psychobiol 47: 111–122. 16136572 10.1002/dev.20082

[48] Miklósi Á, Kubinyi E, Topál J, Gácsi M, Virányi Z, Csányi V (2003) A simple reason for a big difference: wolves do not look back at humans, but dogs do. Curr Biol 13: 763–766. 12725735 10.1016/s0960-9822(03)00263-x

[49] Virányi Z, Gácsi M, Kubinyi E, Topál J, Belényi B, Ujfalussy D, et al (2008) Comprehension of human pointing gestures in young human-reared wolves and dogs. Anim Cog 11: 373–387. 10.1007/s10071-007-0127-y 18183437

